# Rethinking Bias: Positive Healthcare Experiences of Older Gay Men Living With Serious Illness in the Deep South

**DOI:** 10.1093/geroni/igaf122.1812

**Published:** 2025-12-31

**Authors:** Korijna Valenti, Stacy Smallwood, Ronit Elk, Michael Barnett

**Affiliations:** The University of Alabama at Birmingham, Birmingham, Alabama, United States; Wake Forest University School of Divinity, Winston-Salem, North Carolina, United States; The University of Alabama at Birmingham, Birmingham, Alabama, United States; Four Seasons Hospice, Flat Rock, North Carolina, United States

## Abstract

**Background:**

While research on health disparities for sexual and gender minority (SGM) older adults is progressing, less is known about positive experiences for those living with serious illness. This community-based participatory research study examines the perspectives of older cisgender gay men and caregivers from the Deep South, highlighting affirming encounters that challenge dominant narratives of discrimination. Applying Minority Stress Theory and a Queer Gerontology approach, we explore how inclusion, communication, and identity disclosure shape healthcare experiences for older gay men and discuss implications for systemic changes in care.

**Methods:**

Semi-structured interviews were conducted with cisgender gay men (n = 12) aged 50+ living with serious illness and caregivers (n = 4).

**Results:**

Thematic analysis identified three main themes: (1) experiences of inclusion and visibility—meaningful connections with clinicians and compassionate care; (2) positive communication—clear, respectful dialogue enhancing trust and engagement; and (3) sharing sexuality and its effect on care—while disclosure varied, recognition of partnerships improved healthcare interactions. Notably, participants overwhelmingly described positive experiences, thus raising questions about whether discrimination has shifted from overt to more covert forms, or if cultural humility efforts and clinician training may be aiding in successfully improving care.

**Discussion:**

Findings suggest healthcare professionals may be more adept at demonstrating inclusion, yet gaps remain in how systemic biases manifest in subtler ways. Future research should explore whether positive experiences reflect genuine progress, self-selection bias in research participation, or shifting forms of discrimination. Understanding what drives affirming care can inform best practices to ensure all older SGM adults receive equitable, patient-centered care.

